# Mesenchymal stem cell-derived secretomes for therapeutic potential of premature infant diseases

**DOI:** 10.1042/BSR20200241

**Published:** 2020-05-07

**Authors:** Yu Wang, Wei Long, Yan Cao, Jingyun Li, Lianghui You, Yuru Fan

**Affiliations:** 1 Women’s Hospital of Nanjing Medical University, Nanjing Maternity and Child Health Care Hospital, Nanjing 210004, China; 2 Department of Neonatology, Changzhou Maternity and Child Health Care Hospital of Nanjing Medical University, Changzhou 213000, China

**Keywords:** Extracellular vesicles, Mesenchymal stem/stromal cells, Premature infant disease, Secretome

## Abstract

Preterm birth is a complex syndrome and remains a substantial public health problem globally. Its common complications include periventricular leukomalacia (PVL), bronchopulmonary dysplasia (BPD), necrotizing enterocolitis (NEC) and retinopathy of prematurity (ROP). Despite great advances in the comprehension of the pathogenesis and improvements in neonatal intensive care and associated medicine, preterm birth-related diseases remain essentially without adequate treatment and can lead to high morbidity and mortality. The therapeutic potential of mesenchymal stem/stromal cells (MSCs) appears promising as evidenced by their efficacy in preclinical models of pathologies relevant to premature infant complications. MSC-based therapeutic efficacy is closely associated with MSC secretomes and a subsequent paracrine action response to tissue injuries, which are complex and abundant in response to the local microenvironment. In the current review, we summarize the paracrine mechanisms of MSC secretomes underlying diverse preterm birth-related diseases, including PVL, BPD, NEC and ROP, are summarized, and focus is placed on MSC-conditioned media (CM) and MSC-derived extracellular vesicles (EVs) as key mediators of modulatory action, thereby providing new insights for future therapies in newborn medicine.

## Introduction

Preterm birth, defined by the World Health Organization as birth before 37 weeks of pregnancy, is a complex syndrome and remains a substantial global public health problem [1,2]. Approximately 15 million babies are born preterm annually [3], accounting for 11% of all live births worldwide [4]. However, the rate of preterm births varies from 5 to 6% in several European nations to ≥15% in some parts of Africa [4]. In 2013, Guo et al. [5] investigated the birth outcomes of pregnant women from 132 cities in China and found that 7.2% of live births were preterm. The incidence of preterm birth is continuously increasing in many countries [6]. Another study performed by Chawanpaiboon et al. [7] estimated that the global preterm birth rate was between 8.3 and 10.9% in 2000 and between 9.0 and 12.0% in 2014. Additionally, premature labor directly leads to approximately 1 million neonatal deaths annually, contributing to a major cause of childhood morbidity [8].

Severe complications of prematurity include bronchopulmonary dysplasia (BPD), periventricular leukomalacia (PVL), necrotizing enterocolitis (NEC) and retinopathy of prematurity (ROP). The poor outcomes of these diseases have improved over the past decade due to significant advances in neonatal intensive care, especially for premature newborns, and the use of glucocorticoids and pulmonary surfactants. At present, the clinical treatment of premature diseases mainly includes auxiliary ventilation, drug therapy, supportive therapy and surgical treatment [9–11]. For example, the application of glucocorticoid in BPD can inhibit the occurrence of inflammatory reaction, reduce bronchial and pulmonary edema, promote the production of antioxidant enzymes and pulmonary surfactants in the lung, rapidly improve lung function and help to remove the ventilator as soon as possible [12]. However, many studies have reported that glucocorticoid can increase the risk of adverse reactions, such as those by the nervous system, in the early or late stages, and its dose and course of treatment are not certain [13,14]. In terms of surgical treatment, preterm infants with surgical NEC are at risk of significant growth reduction and neurodevelopmental impairment (NDI), including cerebral palsy, deafness and blindness [15,16]. These sequelae and complications of prematurity-related diseases affect not only the neonatal period, but also infancy and childhood, and even adolescence and adulthood [17–19]. Currently, although various treatment methods have been found to achieve certain curative effects, there still exist several side effects and disputes above. Thus, it is necessary to explore and develop new therapies for the treatment and prevention of premature infant diseases.

Accumulating studies have established the importance and feasibility of stem cell-based therapies to ameliorate many degenerative and inflammatory diseases. Among these stem/progenitor cell types, mesenchymal stem/stromal cells (MSCs) have received more extensive attention because they are easily extracted, exert anti-inflammatory effects, have low immunogenicity and can renew themselves. The therapeutic potential of MSCs has been demonstrated in many diseases such as cancer [20,21] and cardiovascular [22,23] and lung [24–26] diseases. MSCs also represent a promising and growing approach to the targeting of various pathological processes and cellular impairments underlying the major abnormalities in premature infant and preterm-associated neonatal diseases [27–29]. Therefore, the mechanisms of MSC action have been hypothesized and researched. Initially, MSCs were thought to work via regenerative effects by homing to injured tissues to engraft and replace damaged cells. However, evidence suggests that the benefits of MSCs are primarily derived from their secretomes and paracrine action [30–33], which exert immunomodulatory, anti-inflammatory, angiogenic, antibacterial and proregenerative effects. This review summarizes the effects of MSC secretomes in diverse preterm birth-related diseases, including PVL, BPD, NEC and ROP, and focuses on MSC-conditioned media (CM) and MSC-derived extracellular vesicles (EVs) as the key mediators of modulatory action, thereby providing new insight for future therapies in newborn medicine.

## Characterization of MSCs and their secretomes

Since MSCs were first reported as being derived from human bone marrow (BM) in 1999 [34], they have been isolated from multiple tissues, including adipose tissue, amniotic fluid, umbilical cord blood, umbilical cord Wharton’s jelly, placental amnion, and placenta [35–37]. Unlike the strong differentiation potential of embryonic stem cells (ESCs) or induced pluripotent stem cells (iPSCs) into various cells from three embryonic germ layers, MSCs can multidifferentiate and be induced into mesenchymal cells, adipose tissue, skeletal muscle cells, bone, and cartilage [38]. The characteristics of MSCs include being plastic-adherent and presenting a typical fibroblast phenotype. Further, they can differentiate into adipocytes, osteoblasts, and chondroblasts *in vitro* under the appropriate conditions and simultaneously express surface antigens such as CD105, CD90, and CD73; however, they lack CD34, CD45, CD14, CD19, and HLA-DR markers [35,36]. Researchers initially assumed that MSCs benefit cells by homing to damaged cells; however, studies gradually found that the transplanted cells either did not differentiate into resident injured cells, or their survival rate was very low [39]. Previous studies have suggested that the MSC-derived cell-free secretomes may be responsible for many of the properties and effects of MSCs. MSC-derived secretomes primarily include bioactive factors and cell-secreted vesicles which can be secreted into extracellular fluid, and then into the blood [40]. These biomolecules can be divided into the following categories, and have protective functions in different tissues: (1) angiogenesis factors, such as vascular endothelial growth factor (VEGF), insulin-like growth factor (IGF-1), and hepatocyte growth factor (HGF); (2) anti-apoptotic factors, such as basic fibroblast growth factor (bFGF), transforming growth factor (TGF), and granulocyte macrophage-colony stimulating factor (GM-CSF); (3) anti-inflammatory factors, such as TNF-a-stimulated gene/protein (TSG), interleukin (IL-10), and heme oxygenase (HO) [41,42]. Additionally, soluble factors such as immunoregulatory protein produced by cells can be found to be sealed in vesicles [43]. These secretomes can mediate diverse functions via a cross-talk between different cell types, and are easily affected by organism environment *in vivo* [44–46]. Many studies have profiled secretomes on the CM and EVs derived from *in vitro* cultures [47] to identify the components responsible for the beneficial effects of MSC-derived secretomes. In the present study, the focus is placed on these two aspects.

### MSC-derived CM

The cell-free CM derived from MSCs is enriched in various soluble factors, including growth factors, immunomodulatory molecules, cytokines, and chemokines [40,48,49]. MSC-derived CM has shown to have beneficial effects both *in vivo* and *in vitro* [50–53]. In addition, preconditioning strategies such as the environmental variations and stimulatory conditions of MSCs can change the composition of CM active factors and influence the CM’s therapeutic effect. Waszak et al. [54] showed that after preconditioning in hyperoxic conditions (95% oxygen), stanniocalcin-1 (STC1) expression significantly increased in MSC-derived CM as compared with that in the normoxic group. Jun et al. [55] revealed that VEGF and TGF β 1 (TGF-β1) expressions increased in either amniotic fluid-derived MSCs (AF-MSCs) or AF-MSC-derived CM in response to hypoxic conditions. Further, hypoxic MSC-derived CM accelerated human dermal fibroblast migration and proliferation compared with that of normal CM [55]. In addition, tumor necrosis factor α (TNF-α)-preconditioned MSC-derived CM has been found to accelerate proliferation, angiogenesis, wound closure, and the infiltration of immune cells into cutaneous wounds *in vivo* [56]. Chemokine, cytokine, and protease secretions are increased in MSC-derived CM after TNF-α-pretreatment, as revealed by proteomic analysis [57]. Bartosh et al. [58] observed that the secretion of anti-inflammatory molecules, namely TNF-α-stimulated gene-6 (*TSG-6*) and STC-1, by MSCs was increased after three-dimensional spheroid culturing, which decreased inflammation *in vivo* and macrophage activation *in vitro*. In addition, TGF-β and interferon-γ (IFN-γ) stimulation [30], the overexpression of angiopoietin-1 [59], HO-1 or other genes [43], and neutralizing antibodies can also change the secretory composition of MSCs and affect their functions.

### MSC-derived EVs: A therapeutic vector of MSCs?

EVs, including exosomes, microvesicles and apoptotic bodies, have been identified as mediators in various physiological and pathophysiological processes [60]. Current studies have revealed that EVs act on target cells by fusing with the target membrane to enter the cell and release the cellular contents, interacting with the receptor–ligand, and inducing the target cells to ingest exosomes via endocytosis [61,62]. Accumulating evidence has shown that MSC-derived EVs effectively restore injured organs and tissues, such as the kidneys, heart, liver, and brain, in animal models, and the substance contained in EVs may play a role in their functioning [60,63,64]. Wang et al. [65] reported that MSC-derived EVs significantly promoted angiogenesis and cardiac function via miR-210 in a mouse myocardial infarction model. Ahn et al. [65,66] explained that the EVs released from MSCs, but not from MSCs with VEGF knockdown, reduced hydrogen peroxide (H_2_O_2_)-induced rat alveolar epithelial cell (L2 cell) death *in vitro* and improved impaired alveolarization, angiogenesis, and inflammatory response in the lungs of hyperoxia-induced rats. This demonstrates that EV-transferred VEGF may be a key paracrine factor that induces protective effects [66]. These observations help explain the therapeutic benefits of EVs in diseases, which may further lead to potential treatments for premature infant diseases.

EVs, once considered cellular debris, range in size from 50 to 200 nm in diameter, are much smaller than cells in diameter (10–30 µm) but larger than proteins, thereby making it difficult to define and isolate them [61]. As analytical technologies for EV detection have improved, investigators have differentiated EVs into three types, namely exosomes, microvesicles, and apobodies, according to their size and morphology [61]. Among them, exosomes have received the most attention from researchers. Exosomes, range in size from 30 to 100 nm in diameter and are membranous particles secreted by MSCs [67]. Mass spectrometry and sequencing analyses have revealed that exosomes secreted by MSCs from different origins share similar constitutions, including DNA, lncRNA, tRNA (transfer RNA), microRNA, and proteins [67,68]. The functions and regulatory pathways of exosome-derived miRNAs and proteins from MSCs have been fully characterized. For miRNAs, Mao et al. [69] demonstrated that exosomes secreted by MSCs that overexpress miR-92a-3p maintained articular chondrocyte functions *in vitro* and reduced cartilage matrix loss in an osteoarthritis (OA) mouse model by decreasing the expressions of collagen type II α 1 (COL2A1), and aggrecan in cartilage. Shi et al. [70] observed that miR-21 was more highly enriched in exosomes derived from H_2_O_2_-treated BM-MSCs as compared with that of normal MSC-derived exosomes. Additionally, Fang et al. [71] demonstrated that UC-MSC-derived exosomes enriched in specific microRNAs (miR-21, miR-23a, miR-125b, and miR-145) played key roles in promoting myofibroblast formation during wound healing. The proteins contained in exosomes also have crucial functions in alleviating organ damage. Wang et al. demonstrated that exosomes secreted by indoleamine 2, 3-dioxygenase (IDO)-overexpressing BM-MSCs (IDO-BM-MSCs) up-regulated the immunoregulatory protein four-and-a-half LIM domain 1 (FHL-1), and that these exosomes reduced the immunoreaction after transplantation and improved cardiac allograft function in rats that received transplants [43]. Yan et al. demonstrated that human umbilical cord MSC (hUC-MSC) exosomes promoted hepatocyte recovery from oxidative stress via the antioxidant and antiapoptotic effects of exosomal glutathione peroxidase1 (GPX1) both *in vitro* and *in vivo*, however, GPX1 knockdown was found to delay this hepatoprotective effect [72]. MSC exosomes have also been reported to mediate several known functions of MSCs in premature infant diseases, including modulating inflammatory/immune responses and reducing fibrosis, apoptosis, and oxidative stress [73–76]. However, more in-depth research is required to explore the mechanisms underlying the effects of exosomes.

## MSC-derived secretomes as a therapeutic strategy for premature diseases

### MSC-derived secretomes for BPD therapy

BPD is a serious long-term complication of preterm birth that leads to significant morbidity and mortality during the neonatal period and is a leading cause of chronic lung disease in children. The incidence of BPD in surviving infants aged ≤ 28 gestational weeks has remained approximately 40% over the last few decades [77–79]. A retrospective study found that the survival rates of infants aged 22–28 gestational weeks has increased from 70 to 79% over the past 20 years in the U.S.A., while the incidence of BPD increased from 32 to 45% [80]. In 2017, in Jiangsu, China, the survival rate for babies with BPD and very low birth weights was 24.9% [81]. However, all present approaches for BPD, including ventilation, oxygen supplementation, fluid intake, nutrition, glucocorticoids, surfactants, vitamin A, and caffeine remain supportive [9]. Thus, further studies are necessary to explore new treatments for BPD, such as cell-based therapy.

Early clinical trials (NCT01297205 [82] and NCT01632475 [83]) have demonstrated the therapeutic effects of MSC engraftment in the treatment of BPD. Accumulating evidence has shown that MSC-derived secretomes may be a potential therapy for BPD (Table 1). MSC-derived CM has also been found to improve BPD-associated vascular injury and parenchyma *in vivo* [54,84–86]. Several studies have indicated that soluble factors, such as macrophage colony-stimulating factor 1, osteopontin [86], and STC1 [54], may be principally responsible for the therapeutic abilities of MSC-derived CM. MSC-derived EV [87] and exosome [73,74,88] treatments also block inflammation and reduce fibrosis and pulmonary vascular remodeling, resulting in improved lung function and the amelioration of pulmonary hypertension in hyperoxia-induced BPD. Willis et al. [73] showed that MSC-derived exosomes modulated the lung macrophage phenotype by suppressing the pro-inflammatory ‘M1’ state and enhancing an anti-inflammatory ‘M2-like’ state both *in vitro* and *in vivo*. Interestingly, another study demonstrated the repair effect of MSC-derived exosomes in a BPD animal model [74]. MSC-derived exosomes can repair the lung injury and improve BPD-associated brain injury as revealed by decreased brain cell death and reversed hypomyelination. TSG-6 has also been detected in MSC-derived exosome fractions and can decrease the pro-inflammatory cytokines, IL-6, TNF-α, and IL-1β; it can also decrease cell death in lungs and reverse BPD-associated cardiac and brain pathologies in BPD mouse models. However, the knockdown of TSG-6 in MSC-derived exosomes abolishes the therapeutic effects of the exosomes [74]. Furthermore, Braun et al. [88] demonstrated that MSC-derived exosomes *in vitro* stimulated the extensive capillary network formation of human umbilical vein endothelial cells (HUVECs) *in vitro*, but this function was diminished by anti-VEGF antibodies. The presence of the VEGF protein has also been revealed in the MSC-derived CM, but not in the exosome-depleted media [88]. Increased comprehension of the MSC-derived secretomes will contribute to the development of new strategies for BPD therapy.

**Table 1:** Summary of therapeutic benefits of MSC secretome in premature diseases

MSCs source	Injury model	Outcomes	Key factor	Reference
Human WJ-MSC	Neonatal mouse BPD-Hyperoxia	Improved pulmonary development ↓Lung fibrosis ↓Loss of peripheral pulmonary blood vessels and peripheral pulmonary arterial remodeling ↑Lung function ↓Inflammation ↓PVH	Exosome	[73]
Human UCMSC and BM-MSC	Neonatal mouse BPD-Hyperoxia	Improved the pulmonary phenotype ↓Pulmonary inflammation and alveolar–capillary leak ↓Loss of peripheral pulmonary blood vessels ↓Pulmonary hypertension and right ventricular hypertrophy ↓Cell death in brain and hypomyelination	Exosome Exosome-TSG-6	[74]
Rat BM-MSC	Neonatal rat BPD-Hyperoxia	Prevented disruption of alveolar growth ↑Lung blood vessel density ↓RVH	Exosome/exosome-VEGF	[88]
Human UC-MSC	Neonatal Rat BPD-Hyperoxia	↑Surface area of alveolar air spaces and the number of alveoli ↓Mean alveolar volume ↓Medial thickness index of small pulmonary vessels	EV	[87]
Mouse BM-MSC	Neonatal mice BPD-Hyperoxia	↓Muscularization of intrapulmonary arterioles Improved lung alveolar architecture ↓Alveolar septal thickness ↓Number of polymorphonuclear cells and macrophages in BALF ↓TNF-α, IL-5 and ↑IL-17 in BALF	CM CM-Opn and Csf1	[86]
Mouse BM-MSC	Neonatal mice BPD-Hyperoxia	↓Parenchymal fibrosis and peripheral PA devascularization ↓Loss of peripheral pulmonary blood vessels ↓Alveolar injury improved lung function ↓Moderate PAH and RVH ↓Peripheral PA muscularization	CM	[84]
Mouse BM-MSC	Neonatal mice BPD-Hyperoxia	Ameliorated parenchymal injury ↑Number of BASCs	CM	[85]
Hyperoxia preconditioned rat BM-MSC	Neonatal rat BPD-Hyperoxia	↓ PAH and RVH ↓ Pulmonary artery medial wall thickness Improved lung architecture	CM CM-STC-1	[54]
IFN-γ preconditioned human UC-MSC	Neonatal rat PVL-LPS	↓IL-6, TNF-α, IL-1β and MCP-1 in the brain ↓MBP-positive area of white matter	CM	[89]
Term UC-MSCs	NPCs	↑GFAP expression profile of NPCs ↑oligodendroglial differentiation in NPCs	CM	[90]
MSCs	NPCs	↑GFAP expression profile of NPCs ↑Olig1 and Olig2 ↓Hes1 ↑Oligodendroglial differentiation in NPCs	CM	[91]
Rat BM-MSC and AF-MSC	Rat NEC-hypertonic feeding and LPS and hypoxia and hypothermia	↓Incidence and severity ↑Intestinal mucosa permeability	Exosome	[92]
Mouse BM-MSC	Neonatal rat NEC-hypertonic feeding and hypoxia and hypothermia	↓Incidence and severity ↑Intestinal mucosa permeability ↑Wound healing in IEC-6 cells *in vitro*	Exosome	[75]
Hypoxic preconditioned human BM-MSC	Mouse ROP-Hyperoxia	↓Retinal ischemia and retinal neovascularization ↑Retinal thickness	Exosome	[76]

Abbreviations: BALF, bronchoalveolar lavage fluid; BASC, bronchioalveolar stem cell; Csf1, macrophage colony stimulating factor 1; GFAP, glial fibrillary acidic protein; Hes1, hairy enhancer of split-1; IEC-6, intestinal epithelial epithelial cells of rat small intestine; MBP, myelin basic protein; MCP-1, monocyte chemoattractant protein-1; NPC, neural progenitor cell; Olig1, oligodendrocyte transcription factor 1; Olig2, oligodendrocyte transcription factor 2; Opn, osteopontin; PA, pulmonary arterial; PAH, pulmonary arterial hypertension; RVH, right ventricular hypertrophy.

### MSC-derived secretomes for PVL therapy

PVL, also called periventricular white matter damage (PWMD), is characterized by a loss of oligodendrocytes and their progenitor cells and is the most common antecedent of cerebral palsy and the main form of brain injury in premature infants [93,940]. Importantly, PVL accounts for 5% of premature births in China [95]. PVL involves focal periventricular necrosis and diffuse gliosis in the surrounding white matter and adversely affects social, adaptive, motor, and cognitive functions in preterm children [96,97]. The factors predisposing infants to PVL mainly include birth trauma, asphyxia, hypoxia–ischemia and immature cerebrovascular development [98]. However, PVL treatment primarily includes symptomatic management and rehabilitation, which cannot effectively improve the PVL prognosis. Therefore, more researchers have focused on the effects of MSCs in PVL therapy.

Zhu et al. [99] demonstrated that hUC-MSCs administered intraperitoneally migrated mainly into the injured cerebral hemisphere in a PWMD model. Importantly, hUC-MSC treatment was found to improve the sensorimotor defects and cognitive behavioral abilities in rats. Moreover, mature oligodendrocyte counts were increased, and the amounts of activated microglia and reactive astrocytes were decreased in the hypoxia–ischemia-induced premature brain [99]; however, whether the paracrine mechanism of the transplanted hUC-MSCs was effective was undetermined. Few studies have focused on MSC secretomes and their regulatory pathway in neonatal brain injury (Table 1). Morioka et al. [89] conducted a cytokine and growth factor array and demonstrated that hUC-MSCs secreted various growth factors and cytokines. These authors found that CM extracted from IFN-γ-pretreated UC-MSCs significantly suppressed IL-1β expression in the brain and reversed brain damage in a lipopolysaccharide-induced PVL-like brain injury rat model [89]. The expressions of anti-inflammatory factors, IDO and TSG-6, in MSCs were simultaneously increased after stimulating IFN-γ and may play an important role in the MSC-derived secretomes [89]. Oppliger et al. [90] found that term UC-MSCs presented a stronger oligodendroglial differentiation effect in neural progenitor cells (NPCs) than in preterm UC-MSCs, and proteome analysis of the CM identified the laminin-α2 subunit (LAMA2) as a potential trigger for the differences. In *in vitro* functional study, LAMA2 knockdown in term Wharton's jelly mesenchymal stem cells (WJ-MSCs) reduced marker gene expression in NPCs co-cultured with WJ-MSCs [90]. Similarly, BM-MSC-derived CM has also been found to induce oligodendroglial differentiation in NPCs [91]. However, because of the difficulty in establishing animal models, the therapeutic benefits of MSC-derived secretomes for PVL and the underlying mechanisms are unclear and should be further investigated to explore more effective treatments.

### MSC-derived secretomes for NEC

NEC is the most common neonatal gastrointestinal emergency, mainly occurring in premature newborns with birth weights less than 1500 g [100]. NEC is treated with surgery and conservative and symptomatic treatment, including fasting with total parenteral nutrition, gastrointestinal decompression and drainage, and fluid balance. Approximately 7–10% of very-low birth weight infants will develop NEC, and, among them, 27–52% will require surgical intervention [101–104]. However, the mortality rate of very-low birth weight infants with surgical NEC is approximately 50% [105]. Surviving infants with surgical NEC have poor neurodevelopmental outcomes and may develop short bowel syndrome and require long-term nutritional management because of malabsorption and growth deficiencies [106–108]. Previously published studies have described primary peritoneal drainage as an alternative treatment to open laparotomy [109]. Moss et al. [110] randomly assigned 117 preterm infants (<34 gestational weeks and birth weight < 1500 g) with NEC complicated with intestinal perforation in a multicenter controlled study; no significant differences in mortality were found between open surgery and primary peritoneal drainage. However, due to the very small sample size, clinically significant differences may have been easily ignored. There are no clear recommendations for clinicians. Other studies also have shown that drug therapy such as postnatal minimal enteral feeding with amniotic fluid, decreased the severity of NEC in pig and mouse models [111–113]. Although a number of studies have been conducted to determine novel preventive and therapeutic interventions [10], large multicenter randomized controlled trials are required for clinical application.

Evidence suggests that MSCs can improve the disease course in experimental NEC [29,114,115], and research on MSC therapy for NEC has been promising. Via histopathological and immunohistochemical evaluation, Tayman et al. [29] and Yang et al. [29] found that MSCs significantly reduced bowel damage severity and improved survival rates in an experimental rat NEC model. Rats with NEC showed significant weight gains and amelioration in their clinical sickness scores after intraperitoneal or intravenous MSC injection. Table 1 summarizes the reported studies on MSC-derived secretomes. McCulloh et al. [92] confirmed that MSC-derived exosomes are equivalent to their derived MSCs in decreasing NEC incidence and severity. Terrence et al. [92] also confirmed that BM-MSC-derived exosomes decreased the incidence and severity of experimental NEC and preserved gut barrier function after NEC exposure *in vivo*. Significantly improved healing rates have been shown in intestinal epithelial cells from rat small intestines (IEC-6 cells) exposed to BM-MSC-derived exosomes [75]. These are important findings, as they support a potential future therapy for this disease that uses MSC-derived exosomes. However, the detailed components of MSC-derived secretomes require identification.

### MSC-derived secretomes for ROP

ROP is a potentially blinding retinal vascular disease that occurs mainly in premature infants, especially in infants of low birth weight (LBW), and is the second-leading cause of childhood blindness in the U.S.A. [116]. Along with the development of perinatal medicine, the survival rates of premature infants and those with LBW have increased, while the prevalence of ROP has increased annually. In the U.K., the incidence of ROP increased in LBW infants from 12.8% in 1990 to 12.5% in 2011 [11]. A collaborative ROP research group enrolled 6091 patients from 22 hospitals in seven administrative regions in China from 2010 to 2012 in a multicenter survey. The overall ROP incidence was found to be 15.2%; there was a 46.9% ROP incidence in babies born at a gestational age of <30 weeks and 30.6% at a birth weight of <1500 g [117]. ROP treatment primarily includes laser photocoagulation therapy, scleral cerclage, vitrectomy, and anti-VEGF treatment [118,119]. Traditional therapy has drawbacks, such as destructiveness and irreversible peripheral vision loss, and the optimal dose of anti-VEGF remains uncertain [120]. The most commonly used dose of anti-VEGF in neonates is half the adult dose, while lower doses have been demonstrated to be effective [120]. However, lower doses require special compounding, and may lead to higher rates of relapse. In addition, it is not clear whether dosing should be decreased with bilateral injections or prior laser or inflammation [120].

The therapeutic effect of MSCs in ROP has been reported in an oxygen-induced retinopathy (OIR) model. Wang et al. [121] and Zhao et al. [122] revealed that the area of retinal neovascularization was significantly smaller in OIR mice with intravitreally injected BM-MSCs on postnatal day 12; the injected MSCs were located in retinal neovascularized areas but did not integrate into the vascular network. Kim et al. [123] showed that human placental amniotic membrane-derived MSCs (AM-MSCs) secreted high levels of TGF-β1 and suppressed endothelial cell proliferation under pathological conditions *in vitro*. Moreover, TGF-β1 siRNA nullified the benefits of MSCs both *in vivo* and *in vitro*; TGF-β1 is a key secretory factor in the regulation of MSC-mediated angiogenesis [123]. Another study described the protective effects of human BM-MSC-derived exosomes against ROP [76]. *In vivo* experiments showed that retinal ischemia severity and the development of retinal neovascularization in the eyes of OIR models were reduced by the intravitreal injection of human BM-MSC-derived exosomes using retinal imaging techniques, fluorescein angiography, and phase-variance optical coherence tomography angiography. Although the exosomes did not completely retard retinal ischemia or neovascularization, the OIR severity was found to be significantly attenuated both qualitatively and quantitatively. Moisseiev et al. [76] also indicated that these human BM-MSC-derived exosomes encapsulated prosurvival-associated proteins from the cAMP response element-binding protein signaling pathway, which is the compensatory response of retinal ischemia. Although the current studies listed in Table 1 explored new approaches to the use of MSC-derived secretomes for the treatment of ROP, the secretome function in ROP and the underlying mechanisms should be further validated.

## Conclusion and future perspectives

The health of premature infants is a complex event, and its related diseases can have continuous and unpredictable adverse effects on infant growth and development, and even on adulthood. Recent data have demonstrated that MSCs have aroused much interest due to their therapeutic effects in neonatology and premature diseases, and this type of therapeutic potential is derived from their secretomes. However, the specific mechanism is not completely understood, and may involve multiple pathways mediated by the secretion of soluble factors and exosomes/microvesicles. In the present study, the development and knowledge of MSC-derived secretomes relevant to neonatology and premature diseases were summarized. Recent reports about the complex components and beneficial effects of MSC-derived secretomes in the premature diseases were also included. Specifically, focus was placed on the therapeutic effects of MSC-derived secretomes, which arise from their capacity to deliver growth factors, immunomodulatory factors, and genetic material to target cells, thereby further promoting the activation of prosurvival and antiapoptotic pathways to enhance tissue repair and regeneration. Thus, MSC-derived secretomes have been demonstrated to display their possible advantages in future regenerative medicine as compared to cell-based therapies.

Based on MSC-derived secretomes, exosomes/microvesicles-based therapies may represent a potential and ideal drug delivery system that provides efficacy for the treatment of premature diseases that lack efficient pharmacotherapies. However, their clinical application and development remain hampered by technological, mechanistic, and safety concerns. Several issues that affect the clinical application of MSC-sourced exosomes/microvesicles must be considered. First, the isolation and characterization of MSC-derived exosomes/microvesicles must be standardized. Second, the immunogenicity of the MSC-derived exosomes/microvesicles remains debatable. Furthermore, various methods adopted to pretreat MSCs via the regulation of their functional properties, such as cytokine priming and hypoxia, present differential effects on the components and therapeutic benefits of MSC-derived secretomes. Finally, more efforts are required to better characterize the biology of MSC-released exosomes/microvesicles, and to fully explore the molecular and epigenetic mechanisms underlying their action of repair on premature diseases, including PVL, BPD, NEC, and ROP.

## Abbreviations

AF-MSC: amniotic fluid-derived MSC
BM: bone marrow
BPD: bronchopulmonary dysplasia
CM: conditioned media
Csf1: macrophage colony stimulating factor 1
EV: extracellular vesicle
GPX1: glutathione peroxidase1
HO: heme oxygenase
hUC-MSC: human umbilical cord MSC
HUVEC: human umbilical vein endothelial cell
IDO: indoleamine 2, 3-dioxygenase
IEC-6: intestinal epithelial epithelial cells of rat small intestine
IFN-γ: interferon-γ
IL: interleukin
LAMA2: laminin-α2 subunit
LBW: low birth weight
MSC: mesenchymal stem/stromal cell
NEC: necrotizing enterocolitis
NPC: neural progenitor cell
OIR: oxygen-induced retinopathy
PVL: periventricular leukomalacia
PWMD: periventricular white matter damage
ROP: retinopathy of prematurity
RVH: right ventricular hypertrophy
STC1: stanniocalcin-1
TGF: transforming growth factor
tRNA: transfer RNA
TNF-α: tumor necrosis factor
TSG: TNF-a-stimulated gene/protein
VEGF: vascular endothelial growth factor
WJ-MSC: Wharton's jelly MSC
